# Structural Covariance Changes of Anterior and Posterior Hippocampus During Musical Training in Young Adults

**DOI:** 10.3389/fnana.2020.00020

**Published:** 2020-05-19

**Authors:** Panfei Guo, Qiongling Li, Xuetong Wang, Xinwei Li, Shaoyi Wang, Yongqi Xie, Yachao Xie, Zhenrong Fu, Xiaohui Zhang, Shuyu Li

**Affiliations:** ^1^School of Biological Science and Medical Engineering, Beihang University, Beijing, China; ^2^Beijing Advanced Innovation Center for Biomedical Engineering, Beihang University, Beijing, China; ^3^State Key Laboratory of Cognitive Neuroscience and Learning & IDG/McGovern Institute for Brain Research, Beijing Normal University, Beijing, China; ^4^Center for Collaboration and Innovation in Brain and Learning Sciences, Beijing Normal University, Beijing, China

**Keywords:** anterior hippocampus, posterior hippocampus, structural covariance, musical training, partial least squares

## Abstract

Musical training can induce the functional and structural changes of the hippocampus. The hippocampus is not a homogeneous structure which can be divided into anterior and posterior parts along its longitudinal axis, and the whole-brain structural covariances of anterior (aHC) and posterior hippocampus (pHC) show distinct patterns in young adults. However, little is known about whether the anterior and posterior hippocampal structural covariances change after long-term musical training. Here, we investigated the musical training-induced changes of the whole-brain structural covariances of bilateral aHC and pHC in a longitudinal designed experiment with two groups (training group and control group) across three time points [the beginning (TP1) and the end (TP2) of 24 weeks of training, and 12 weeks after training (TP3)]. Using seed partial least square, we identified two significant patterns of structural covariance of the aHC and pHC. The first showed common structural covariance of the aHC and pHC. The second pattern revealed distinct structural covariance of the two regions and reflected the changes of structural covariance of the left pHC in the training group across three time points: the left pHC showed significant structural covariance with bilateral hippocampus and parahippocampal gyrus, left calcarine sulcus only at TP1 and TP3. Furthermore, the integrity of distinct structural networks of aHC and pHC in the second pattern significantly increased in the training group. Our findings suggest that musical training could change the organization of structural whole-brain covariance for left pHC and enhance the degree of the structural covariance network differentiation of the aHC and pHC in young adults.

## Introduction

Brain plasticity refers to the ability of our brain to modify its organization to learn new skills and adapt to new environments (Draganski et al., 2004; Kehayas and Holtmaat, 2017). It comes with structural changes in GMV (Koch et al., 2016) and cortical thickness (Hervais-Adelman et al., 2017), and functional changes in the intensity of neural responses (Sonntag and Arendt, 2019) and the number of activated voxels in specific tasks (Thompson et al., 2016). Musical training involves multiple sensory modalities and cognitive processes, which makes it an ideal model to investigate training-induced brain plasticity (Herholz and Zatorre, 2012; Gujing et al., 2019). Previous studies suggested that intensive musical training could lead to structural and functional changes in the human brain. For example, playing a musical instrument could lead to a more rapid cortical thickness maturation within areas associated with motor planning and coordination, visuospatial ability, emotion and impulse regulation (Hudziak et al., 2014). What’s more, increased functional connectivity between the cerebellum and hippocampus is observed in musicians compared to non-musicians (Burunat et al., 2018).

The hippocampus is a three-layered cortical structure at the border of the neocortex (Michael, 2011). It plays a key role in learning and memory (Zhan et al., 2018; Crestani et al., 2019), as well as spatial representation (Strange et al., 2014), and is closely related to musical training activities. Previous studies have shown that musical training leads to supplementary activations in the hippocampus during a musical familiarity task and higher gray matter density of the hippocampus in musicians (Groussard et al., 2010). Enhanced neural responses to temporal novelty of sounds were also shown in the left aHC of professional musicians (Herdener et al., 2010). Additionally, the hippocampus is not a homogeneous structure that can be divided into two parts (aHC and pHC) along its longitudinal axis (Poppenk et al., 2013). It is different in the dentate gyrus proportion (Malykhin et al., 2010) and choroid plexus coverage (Duvernoy, 2005) between aHC and pHC in human beings. The neural pathways that connect aHC and pHC to the neocortex and other subcortical regions are also distinct (Fanselow and Dong, 2010). Meanwhile, functional specialization along longitudinal axis has also been observed in the human hippocampus (Poppenk et al., 2013; Strange et al., 2014). Furthermore, previous studies confirmed that a contrast of aHC and pHC structural covariance was significant at the whole-brain level in young adults (Persson et al., 2014; Stening et al., 2017). Together, all of the above findings indicate that considering the aHC and pHC separately is necessary when exploring the influence of musical training activities on hippocampal structural covariance. Nevertheless, to our knowledge, it remains largely unexplored whether hippocampal structural covariance changes after musical training along its longitudinal axis in young adults.

Partial least squares method (Krishnan et al., 2011) is a multivariate statistical technique in which seed PLS can be used to identify LVs relating the seed region (s) and the whole brain across participants in an optimal way, as well as indicating patterns of structural covariance (Nordin et al., 2018). There are several advantages of PLS: First, PLS overcomes a limitation of mass-univariate approaches by increasing the sensitivity to detect subtle or spatially distributed effects in brain signals (McIntosh and Lobaugh, 2004). Second, the decomposition (i.e. singular value decomposition) and associated resampling techniques (i.e. permutation test and bootstrap test) enclosed in the PLS consider all voxels simultaneously, avoiding the problem of multiple statistical comparison (Spreng and Turner, 2013). Third, in contrast to the very similar canonical correlation analysis method, the coefficients derived from PLS are easier to interpret and more stable (Ziegler et al., 2013). Using the seed PLS method in young adults, previous studies revealed distinct structural covariance patterns of the aHC and pHC between men and women (Persson et al., 2014), and a structural covariance pattern of the pHC in Apolipoprotein E (APOE) ε4 carriers was demonstrated to be different to the aHC in non-carriers (Stening et al., 2017).

Overall, musical training could induce structural and functional plasticity in the hippocampus. However, there is limited knowledge for the effects of musical training on the whole-brain structural covariances of the hippocampus. Previous studies confirmed that hippocampal structural covariances were different between aHC and pHC, and enhanced neural responses to temporal novelty of sounds were only shown in the left aHC (not in the pHC) of professional musicians (Herdener et al., 2010). Therefore, we assumed that the structural plasticity induced by musical training are different along hippocampal longitudinal axis. Here, we used seed PLS to assess the potential changes of the whole-brain structural covariance of bilateral aHC and pHC induced by musical training, and also assessed the training effects on hippocampal volume. It is supposed that musical training could lead to changes of hippocampal structural covariance in the training group while no changes would occur in controls. Additionally, we examined to what degree the hippocampal structural covariance patterns varied with musical training along the longitudinal axis. Identifying potential changes in hippocampal structural covariance may contribute to explaining the mechanism of brain plasticity induced by musical training.

## Materials and Methods

### Participants

Sixty young adults (29 males and 31 females) aged between 20 to 26 years old were recruited from students at Beihang University for the present study. All subjects had no history of brain injury, neurological disease or other serious medical conditions. Depressive persons, which were identified by a score > 14 on the BDI (Wang and Gorenstein, 2013), were excluded. All participants were identified as being right-handed based on the handedness questionnaire (modified version of the Edinburgh Handedness Inventory) (Oldfield, 1971) and Chinese was their native language. They were all provided with written informed consent forms before the protocol-specific procedures. Our research was approved by the local ethics committee.

### Assessment

Advanced Measures of Music Audiation, developed by Edwin E. Gordon, was performed on each participant to measure musical aptitude or the potential to learn in the musical domain. IQ scores including performance IQ and verbal IQ of all participants were tested according to the Wechsler Adult Intelligence Scale-Revised Chinese revised version (WAIS-RC) (Gong, 1992). The participants, who lacked a musical background (AMMA score: 20∼80) and had normal intelligence (IQ score: <140), were divided into a training group (30 participants) and a control group (30 participants) randomly. Four participants (three males and one female) dropped out of the experiment due to the failure to comply with the training rules or other health problems irrelevant to the study design. As a result, the training group and the control group composed of 29 participants (13 males) and 27 participants (13 males), respectively, in the end (see Table 1 for subject characteristics).

**TABLE 1:** The participant demographic and characteristics at baseline in the control group and training group (SDs in parentheses).

	Control (*N* = 27)	Training (*N* = 29)	*p*
Age, yrs	23.33 (1.39)	23.10 (1.37)	0.536
Gender,M/F	13/14	13/16	0.803
Education,yrs	16.70 (1.26)	16.59 (1.09)	0.709
BDI	5.15 (3.87)	4.71 (3.62)	0.646
IQ	128.19 (7.33)	129.11 (5.65)	0.598
AMMATonalpercentilerank	54.26 (14.78)	59.34 (10.78)	0.145
AMMARhythmpercentilerank	46.90 (14.54)	53.83 (11.61)	0.116
AMMACompositepercentilerank	51.93 (15.31)	57.28 (11.08)	0.351

*Two sample *t*-tests are implemented for these data (except for gender), Chi-square test is performed for gender. No significant differences are observed between these two groups. Note: yrs, years; M/F, Male/Female.*

We used a within-subject design: the training group received a 24-week musical training including professional instructions, scheduled practice and a final musical performance. On the other hand, the participants in the control group were asked not to get involved in any musical training throughout the period of our study. Participants were all tested at three time points: (TP1, the beginning of musical training), (TP2, the end of musical training) and (TP3, 12 weeks after TP2). At each time point, all participants received Magnetic Resonance Imaging (MRI) scanning sessions and a set of behavioral tests: three subtests (block design, digit symbol, and digit span, measuring the spatial and sequential memory) of the WAIS-RC, two subtests (part A and B) of trail making test (Lezak et al., 2004) (measuring the processing and motor abilities).

A two-sample *t*-test implemented in SPSS (SPSS version 19) was used to test the participant demographic and characteristics at baseline, except for gender (Chi-squared test). Two-sample *t*-tests were also performed to examine the cognitive abilities at baseline. Mixed-ANOVAs were conducted to assess the main effect of time and the interaction effect of time over group on cognitive abilities, in which time (i.e. Tp1, Tp2, and Tp3) was treated as within-subject factor and group (i.e. training group and control group) was treated as between-subject factor, including age, gender, and education as covariates of no interest. Significant interactions were followed by *post hoc* pair *t*-tests between the factor of time to determine which time point differ from each other.

### Procedures

Participants in the training group were given instructions on musical theory, musical performance as well as technique exercises 1 h per week in the form of one-to-two musical lessons taught by professional musicians. The musical theory taught in the weekly 1 h course took about 10 min and the time of the musical theory learning was nearly the same for each participant (about 4 h) for the whole training program. A typical course began with error correction in the weekly musical theory assignment and interpretation of new musical theory of study. The teaching of musical theory and musical performance was under the guidance of the Bastien Piano for Adults-Book 1 (Bastien et al., 2000). From the beginning of the 18th week, in addition to the exercises in Bastien, one technical motor exercise according to Hanon Piano Fingering Practice, was assigned weekly due to requiring 1 week for participants to complete at a moderate tempo. It should be noted that a minimum practice time of five 30 min sessions (i.e. 5 days, at least 30 min practice per day) and a maximum practice time of seven 60 min sessions (i.e. 7 days, at most 1 h of practice per day) each week in the assigned room was required, and the practice time was logged (6483.8 ± 1061.3 min). At the end of the training, the participants played selected pieces from Bastien Piano for Adults-Book 1 and their performance was evaluated by professional musicians: each participant played the selected piece individually and skillfully, which indicated their technical ability equivalent to those with certifications verified by the Central Conservatory of Music piano level 4.

### MRI Acquisition

Scanning was performed on a SIEMENS Trio Tim 3.0 T scanner with a 12-channel phased array head coil in the Imaging Center for Brain Research, Beijing Normal University. We used the 3D high-resolution brain anatomical T1-weighted images in this study, obtained with a sagittal 3D Magnetization Prepared Rapid Gradient Echo sequence. Sample acquisition parameters were as follows: repetition time = 2530 ms; echo time = 3.39 ms; inversion time = 1100 ms; flip angle = 7°; field of view = 256 × 256 mm^2^; in-plane resolution = 256 × 256; slice thickness = 1.33 mm; number of sagittal slices covering the whole brain = 144; isotropic resolution = 1.33 × 1 × 1 mm^3^.

### Preprocessing

All structural T1-weighted images were preprocessed using the Computational Anatomy Toolbox 12 (CAT12^1^) in Statistical Parametric Mapping 12 (SPM12^2^) implemented with MATLAB R2012a (The Mathworks, Natick, MA). The T1-weighted structural images were processed using the longitudinal preprocessing module in CAT12, which was developed and optimized for detecting more subtle effects over shorter time ranges, as well as significantly increase reliability and statistical power in longitudinal studies (Reuter et al., 2012). As a result, we obtained gray matter images normalized to the Montreal Neurological Institute (MNI) space. It should be noted that in order to preserve regional volume information at spatial normalization, modulation of the normalized gray matter images should be done before the final smooth with a Gaussian kernel of 8 mm Full Width at Half Maximum (FWHM). Additionally, quality checks of the images were performed before and after the preprocessing to safeguard against influence of other factors such as noise, motion of the images and misregistration.

Considering individual differences in brain size, the TIV of each person was subsequently used to scale the values of voxels in the preprocessed gray matter images. Therefore, voxel value used for subsequent analysis was equal to the corresponding voxel value in smoothed image divided by TIV, which represented proportional regional GMV.

### Volumetric of Anterior and Posterior Hippocampus

**FIGURE 1:**
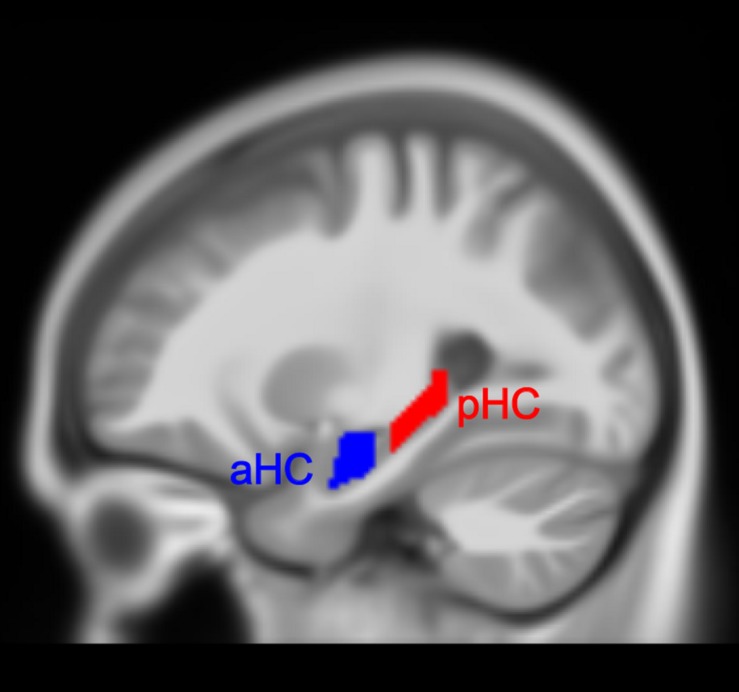
Anterior (aHC) and posterior (pHC) division of the hippocampus.

The hippocampal label was taken from the Automated Anatomical Labeling (AAL) atlas (Rolls et al., 2015) from the Wake Forest University PickAtlas (WFUPickatlas) toolbox (Maldjian et al., 2003^3^) and then superimposed onto the MNI152 T1 template involved in the CAT12 toolbox. We utilized the appearance of the uncal apex on coronal slices to divide the hippocampus into anterior and posterior parts, based on a definition of the aHC and pHC (Poppenk et al., 2013). Of note, some researchers also divided the hippocampus into the hippocampal head, body, and tail (Malykhin et al., 2010). The aHC in our study corresponded to the hippocampal head, and pHC corresponded to the hippocampal body and tail. Additionally, we removed a 2 mm coronal slice from each of the two adjacent ends to avoid contamination between the regions which might be induced by misregistration or partial volume effects. The final definitions of the aHC and pHC spanned from -2 to -18 and -24 to -42 along the y-axis separately in MNI space (Persson et al., 2014; Nordin et al., 2018) (see Figure 1 for the view of aHC and pHC). As a result, we obtained four hippocampal subregions: left aHC, left pHC, right aHC and right pHC.

For each individual TIV-scaled gray matter image, the voxel values belonging to the respective subregion were summed and divided by the total number of voxels to quantify the average voxel value of that region. The average voxel values here represent regional volume information of the region of interest (ROI) and are used for further analysis. Mixed-ANOVAs were conducted to assess the main effect of time and the interaction effect of time over group on TIV, GMV, and average voxel values of four hippocampal subregions, in which time (i.e. Tp1, Tp2, and Tp3) was treated as a within-subject factor and group (i.e. training group and control group) was treated as a between-subject factor, including age, gender, and education as covariates of no interest. Significant interactions were followed by *post hoc* pair *t*-tests between the factor of time to determine which time points differ from each other.

### Structural Covariance of the Bilateral Anterior and Posterior Hippocampus: Seed Partial Least Squares

Seed PLS^4^ (McIntosh and Lobaugh, 2004; Krishnan et al., 2011) has been commonly used to identify LVs indicating patterns of structural covariance between the volume of seed (i.e. ROI) and the global brain. In each LV, every participant has a brain score which is calculated to measure how strongly the covariance pattern is expressed in that particular participant. Meanwhile, each voxel has a positive or negative salience that represents its contribution to the pattern described by the LV. In term of structural seed PLS, a brain score for each participant can be mathematically expressed as a dot product of the gray matter voxel value in the normalized segmented image and the corresponding voxel salience for each LV.

Here, we assessed the structural whole-brain covariance of the HC across three time points using one PLS analysis. Seed-region values (i.e. average voxel values of four hippocampal subregions) and the structural brain gray matter images of all participants across three time points were all in one time entered into the PLS analysis, ordered according to sub-group (i.e. control group and training group) where each group have three conditions (i.e. TP1, TP2, TP3). The statistical significance of the LVs is estimated using 2000 permutations, in which significant threshold is set at *p* < 0.05. The reliability of the voxel included in these LVs was calculated by 1000 bootstrapping procedures and expressed as a voxel-wise (BSR; the ratio of the salience to the bootstrap standard error). We identified the threshold of BSRs at a conservative value of ± 3.8 to obtain voxels with high reliability (i.e. voxels with BSR > 3.8 or BSR < −3.8 were considered reliable, corresponding to a *p*-value of 0.0001). As seed PLS is a multivariate method executed in a single analytic step, no corrections for multiple comparisons were needed (Persson et al., 2014; Nordin et al., 2018).

## Results

### Demographics and Volumes

The training group and control group had no significant difference in age, gender, level of education, BDI, IQ, and all AMMA scores (*p* > 0.05, see Table 1) at the baseline. Performing FDR control (Glickman et al., 2014), the groups did not show significant difference in TIV, GMV, average voxel values of four hippocampal subregions and all cognitive scores at baseline (*p* > 0.05, see Table 2), and neither significant time effect nor interactions of the group over time effects were found for TIV, GMV, average voxel values of four hippocampal subregions, trail making test A and B, Block design, Digit span, and Digit symbol. Additionally, because no significant time effect, as well as the time over group effect were found for all volumetric measures (i.e. TIV, GMV, average voxel values of four hippocampal subregions) and cognitive scores, we did not involve them in correlation analysis with the practice time.

**TABLE 2:** TIV, GMV, average voxel values of four hippocampal subregions and all cognitive scores in the control group and training group across three time points (SDs in parentheses).

	Control			Training			
		
	TP1	TP2	TP3	TP1	TP2	TP3	*P*
**Volumes**							
TIV (mm^3^)	1550 (123)	1550 (124)	1550 (125)	1519 (126)	1517 (125)	1518 (125)	0.651
GMV (mm^3^)	737.2 (53.2)	733.9 (51.8)	735.1 (52.1)	716.6 (51.8)	714.9 (51.5)	714.9 (54.8)	0.651
Left aHC	0.392 (0.027)	0.391 (0.028)	0.393 (0.027)	0.390 (0.026)	0.390 (0.026)	0.390 (0.025)	0.945
Left pHC	0.278 (0.020)	0.277 (0.021)	0.277 (0.020)	0.279 (0.024)	0.277 (0.024)	0.278 (0.023)	0.945
Right aHC	0.368 (0.023)	0.366 (0.023)	0.367 (0.023)	0.362 (0.024)	0.362 (0.025)	0.362 (0.024)	0.651
Right pHC	0.258 (0.023)	0.259 (0.022)	0.257 (0.021)	0.264 (0.027)	0.262 (0.027)	0.263 (0.026)	0.651
**Cognitive scores**							
TMT-A	23.37 (5.90)	19.94 (4.39)	17.95 (3.15)	27.76 (10.28)	24.10 (8.95)	21.32 (8.15)	0.627
TMT-B	54.75 (16.68)	43.43 (9.25)	47.45 (22.92)	60.11 (35.04)	45.18 (12.55)	41.94 (13.72)	0.652
Block design	14.15 (1.23)	14.63 (0.97)	14.48 (0.98)	14.14 (1.22)	14.52 (0.95)	14.72 (0.75)	0.975
Digit span	15.19 (2.50)	15.70 (2.27)	16.07 (2.30)	14.48 (2.52)	15.62 (2.48)	16.28 (2.27)	0.651
Digit symbol	17.07 (1.59)	17.44 (1.40)	17.59 (1.25)	17.48 (1.57)	18.07 (1.10)	18.21 (1.05)	0.651

*Two sample *t*-tests are implemented, and FDR control is executed subsequently. *P* values are all FDR-corrected in this table. *P* < 0.05 is considered significant. Note: Left aHC, average voxel value of left anterior hippocampus; Left pHC, average voxel value of left posterior hippocampus; Right aHC, average voxel value of right anterior hippocampus; Right pHC, average voxel value of right posterior hippocampus; TMT-A, trail making test A; TMT-B, trail making test B.*

### Structural Covariance of Bilateral Anterior and Posterior Hippocampus

**FIGURE 2:**
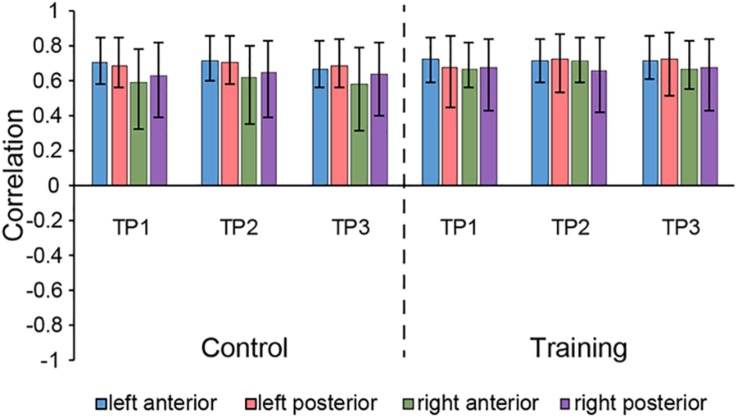
Correlations between brain scores and seed-region volumes in LV1, showing similar covariance of the bilateral aHC and pHC comparable within each group across three time points. Bars describe the relation of each seed to the structural covariance pattern. The error bars represent 95% bootstrapped confidence intervals. A lack of overlap in interval indicates reliable difference for any two correlations and overlap with 0 means the correlation is not significantly different from 0.

The seed PLS analysis yielded two significant LVs (i.e. LV1 and LV3) representing the structural covariance patterns of the hippocampus. LV1 (*p* < 0.001; accounting for 41.83% of the cross-correlation variance) captured whole-brain covariance common to the aHC and pHC. The aHC and pHC of both groups were significantly related to this pattern and show similar seed-correlations across three time points (see Figure 2 for the correlations between seed regions and the covariance pattern). The pattern captured by LV1 included positive covariance of the bilateral aHC and pHC with clusters extended through the entire length of the bilateral hippocampus, and to the parahippocampal gyrus, thalamus, amygdala, left inferior parietal lobule, and the right inferior frontal gyrus. Negative covariance was found in the left precuneus (see Table 3 for the report of all reliable clusters).

**TABLE 3:** Clusters of reliable voxel saliences for latent variable 1, ordered from the most anterior to the most posterior.

Location	Voxel	MNI coordinates			BSR
		
		*X*	*Y*	*Z*	
**Positive saliences**					
Inferior frontal gyrus (R)	156	58.5	28.5	22.5	5.081
Insula (R)	63	43.5	6	–12	4.301
Precentral gyrus (L)	70	–49.5	–9	6	4.542
Parahippocampal gyrus ^a^ (L)	7265	–25.5	–18	–21	12.896
Supplementary motor area (R)	51	3	–18	52.5	4.825
Inferior parietal lobule (L)	183	–43.5	–34.5	28.5	4.272
Parahippocampal gyrus ^b^ (R)	6913	19.5	–37.5	4.5	10.428
Cerebellum (R)	82	33	–55.5	–63	4.088
Cerebellum (L)	195	–33	–66	–54	4.455
Superior occipital gyrus (R)	60	27	–85.5	24	4.437
Calcarine sulcus (L)	254	–12	–87	10.5	5.403
**Negative saliences**					
Precuneus (L)	60	–7.5	–58.5	43.5	-4.379

*^*a*^represents this cluster also includes ipsilateral hippocampus, thalamus, amygdala, lingual gyrus, and superior temporal gyrus. ^*b*^represents this cluster also includes ipsilateral hippocampus, thalamus, amygdala, and superior temporal gyrus. Coordinates and BSR of peak voxel are reported for each cluster. Voxels with BSR > 3.8 or BSR < −3.8 are considered to be reliable, and clusters exceeding 50 voxels are reported. Note: L, left; R, right.*

**FIGURE 3:**
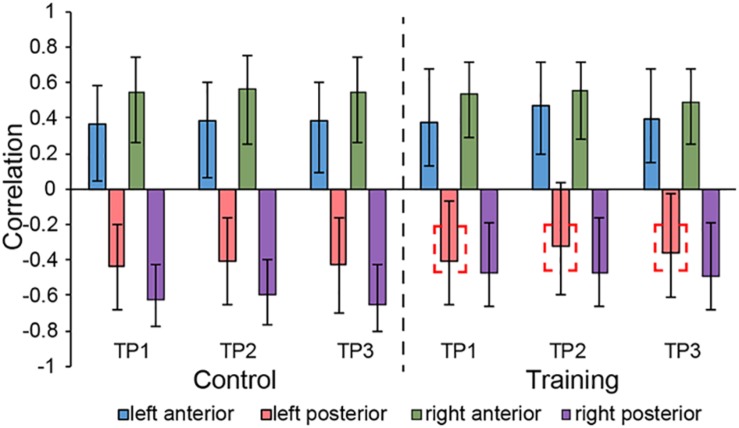
Correlations between brain scores and seed-region volumes in LV3, showing a special structural covariance pattern of aHC in both group across three time points, which is different from the covariance pattern of pHC. For training group, the correlation of left pHC was only significant at TP1 and TP3 other than the TP2 and the red dotted box is used for indicating the left pHC across three time points. Bars describe the relation of each seed to the structural covariance pattern. The error bars represent 95% bootstrapped confidence intervals. A lack of overlap in interval indicates reliable difference for any two correlations and overlap with 0 means the correlation is not significantly different from 0.

LV3 (*p* < 0.001; accounting for 14.16% of the cross-correlation variance) revealed the distinct structural covariance patterns of the aHC and pHC (see Figure 3 for the correlations between seed regions and the covariance pattern). The aHC showed significant positive covariance with the bilateral anterior temporal lobe, and the right inferior parietal lobule and superior temporal gyrus. Also reflected in this LV, the pHC showed significant positive covariance with the bilateral posterior temporal lobe, and the left calcarine sulcus (see Table 4 for the report of all reliable clusters). On the other hand, among all seed regions within both groups, LV3 only reflected the changes of structural covariance of the left pHC in the training group, which was made evident by correlations of left pHC only being significant at TP1 and TP3 across three time points. Additionally, the aHC and pHC further displayed negative covariance with each other, suggesting a division also in intra-hippocampal covariance.

**TABLE 4:** Clusters of reliable voxel saliences for latent variable 3, ordered from the most anterior to the most posterior.

Location	Voxel	MNI coordinates			BSR
		
		*X*	*Y*	*Z*	
**Positive saliences**					
Superior temporal gyrus (R)	103	34.5	9	-49.5	5.800
Anterior temporal lobe ^a^ (L)	1007	–22.5	–9	-25.5	6.168
Anterior temporal lobe ^a^ (R)	1034	22.5	–10.5	-24	5.583
Inferior temporal gyrus (L; extending into middle temporal gyrus)	259	–61.5	–13.5	-25.5	4.296
Inferior parietal lobule (R)	62	43.5	–54	45	4.687
**Negative saliences**					
Posterior temporal lobe ^b^ (R)	1391	31.5	–39	-3	-6.605
Posterior temporal lobe ^b^ (L)	591	–28.5	–42	0	-5.532
Calcarine sulcus (L)	101	3	–91.5	7.5	-4.639

*^*a*^represents this cluster covers the hippocampus, parahippocampal gyrus, amygdala. ^*b*^represents this cluster covers the hippocampus, parahippocampal gyrus, thalamus. Coordinates and BSR of peak voxel are reported for each cluster. Voxels with BSR > 3.8 or BSR < −3.8 are considered to be reliable, and clusters exceeding 50 voxels are reported. Note: L, left; R, right.*

### Brain Scores of Control Group and Training Group

**FIGURE 4:**
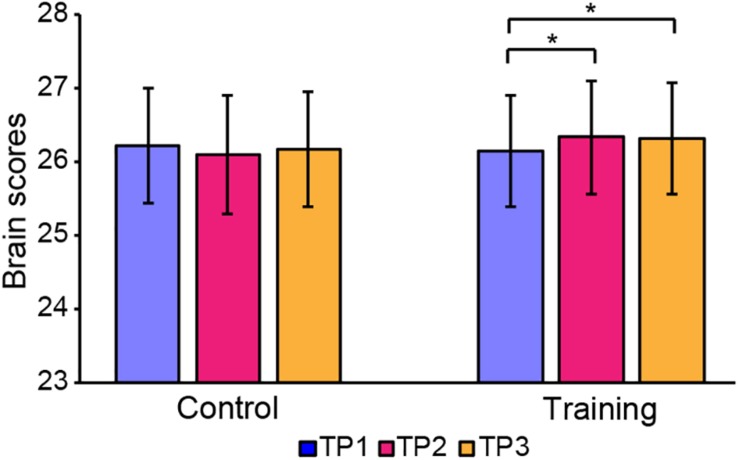
Brain scores of latent variable 3 in the control group and training group across three time points. * represents *p* < 0.05.

The brain scores of the control group and training group at baseline were not significantly different for both LV1 (*p* > 0.05, 79.78 ± 4.71 and 78.99 ± 4.44, respectively) and LV3 (*p* > 0.05, 26.27 ± 4.69 and 26.10 ± 3.47, respectively). Mixed-ANOVAs were also performed for the brain scores of both groups across three time points in LV1 and LV3, respectively. Significant interaction of the group over time was shown only in LV3 (*F* = 4.654, *p* = 0.012). For LV3, the subsequent *post hoc* pair *t*-tests revealed that brain scores both in TP2 and TP3 were significantly greater than TP1 in the training group, while no significant differences were found between any two time points in the control group (see Table 5 and Figure 4).

**TABLE 5:** *Post hoc* pair *t*-tests results for brain scores of latent variable 3 in the control group and training group across three time points.

	Control		Training	
		
	Δ Brain scores	*p*-value	Δ Brain scores	*p*-value
TP2 vs. TP1	−0.123	0.170	0.190	0.029*
TP3 vs. TP2	0.067	0.300	−0.009	0.882
TP3 vs. TP1	−0.056	0.472	0.182	0.017*

*represents *p* < 0.05. The “Δ Brain scores” is equal to the mean value of previous time point minus the following time point.

## Discussion

In this study, no volumetric (i.e. TIV, GMV, average voxel values of four hippocampal subregions) differences were found between the training group and control group. Considering the limited brain plasticity for young adults and the limited training duration, this may be due to the fact that the structural changes during our study are relatively subtle and difficult to detect using global methods. Therefore, we used the seed PLS approach which has been proven to increase the sensitivity to detect subtle or spatially distributed effects in brain signals (McIntosh and Lobaugh, 2004), to examine the potential hippocampal structural covariances changes induced by musical training. We obtained two significant LVs (LV1 and LV3) in young adults where the LV1 showed a similar structural covariance pattern for bilateral aHC and pHC, while the LV3 revealed distinct structural covariance patterns of the aHC and pHC, as well as reflected the changes of structural covariance for left pHC across the training process. Also reflected in LV3, the integrity of distinct structural networks of aHC and pHC significantly increased in the training group. Our study provides evidence for structural connectivity changes of the hippocampus related to musical training in young adults along its longitudinal axis.

The LV1 display a structural covariance pattern common to the aHC and pHC. Several of the regions co-varying with the whole hippocampus in the present study are regions of importance for memory and cognition. Frontal gyrus and occipital gyrus are involved in hippocampal-whole brain functional connectivity supporting memory (Ranganath et al., 2005). This covariance pattern also extended to the insula and cerebellum which are important for cognition (Chang et al., 2013; Koziol et al., 2014). These findings suggest that the structural covariance pattern observed here might be related to memory and cognition, which have already been widely reported as the main function of the hippocampus. Musical training involved large amounts of memory and cognition-related activities (e.g. musical theory learning), which might explain the reason why the result of LV1 accounts for the major cross-correlation variance (i.e. 41.83%). On the other hand, regions showing positive covariance with the hippocampus in LV1 were highly consistent with the early findings (Persson et al., 2014; Nordin et al., 2018), which also supported the reliability for the structure covariance pattern of the hippocampus represented in LV1.

The LV3 discriminated the structural covariance pattern between aHC and pHC. Previous research revealed a classical proposition: HIPER (Hippocampal Encoding/Retrieval) model (Lepage et al., 1998), suggesting a division of memory-related labor between the rostral and caudal portions of the hippocampal formation (i.e. activations in the hippocampal region associated with memory encoding are located primarily in the rostral portions of the region, whereas activations associated with memory retrieval are located primarily in the caudal portions). The HIPER model supported the structural covariance pattern in LV3. As we can see in Table 4, several of the regions within the structural covariance pattern of aHC observed here play a key role in information encoding. The parahippocampal gyrus is important for signal transmission to the hippocampus, and together with the aHC contribute to encoding and item memory (Duvernoy, 2005; Zeidman and Maguire, 2016). Moreover, the amygdala is involved in learning-related coding activities (Rudebeck et al., 2017) and the anterior temporal lobe has also been confirmed to play an important role in semantic memory (Rice et al., 2018). The above findings suggest that this anterior hippocampal covariance pattern may be related to information encoding, which is important for participants to know well, with the musical staff involved in the training process. In contrast, within the structural covariance pattern of pHC, the posterior parahippocampal gyrus is related to the spatial layout of local scene (Huntgeburth et al., 2017), and together with the pHC, are involved in spatial cognition and navigation (Poppenk et al., 2013). Calcarine sulcus are also related to spatial behavior (Liu et al., 2017). Therefore, we might speculate that the structural covariance pattern of the pHC observed here was associated with the spatial ability, relating to the large amount of finger exercises in our musical training design. These conclusions are also consistent with previous studies suggesting that the anterior and posterior hippocampal function correspond to information coding and spatial cognition, respectively (Strange et al., 2014). Additionally, the aHC and pHC displayed negative covariance with each other, further confirming that the hippocampus is not a homogeneous structure that can be divided into two parts along its longitudinal axis at the level of intra-hippocampal covariance.

In addition, for the musical training group, we merely observed changes in structural covariance of left pHC in LV3, which at TP2 was not significantly related to the covariance pattern as it was at TP1 and TP3, but the right pHC remained constant across the three time points. This phenomenon supported the point of functional lateralization of the hippocampus (Robinson et al., 2016). Studies have demonstrated that episodic and spatial memory show functional lateralization (Persson et al., 2013; Ezzati et al., 2016), where the right hippocampus appears particularly related to memory of locations, and the left hippocampus is more involved in context-dependent episodic memory. Combining previous studies which demonstrated the aHC and pHC individually corresponded to episodic and spatial memory, the lateral effect of the pHC shown in LV3 is likely because the left pHC is less related to spatial behavior than the right pHC. Previous studies suggested that a significant portion of structural covariation networks could be attributed to the simultaneous development of different brain regions (Alexander-Bloch et al., 2013). Therefore, when being affected by the musical training which enclosed large amounts of finger exercise, consisting of precise spatial navigation to help participants know well with the corresponding position of each syllable, the left pHC was not consistently coactivated with other regions involved in the network as the right pHC. However, with the cessation of musical training at TP2, the brain regions in the covariance network of left pHC continuously played a synergistic role in spatial-related daily activities. Therefore, 12 weeks after the end of musical training (i.e. at TP3), this whole-brain structural covariance pattern of the left pHC was restored.

Of note, the brain scores of LV3 at TP2 and TP3 were significantly greater than TP1 in the musical training group, indicating that participants’ expression of both anterior and posterior hippocampal covariance patterns in LV3 could be significantly strengthened by musical training. As we mentioned above, aHC plays a key role in information encoding and memory while pHC corresponds to spatial navigation. The regions of the anterior hippocampal covariance network could be activated by musical theory learning activities related to information encoding and memory, while long-time finger exercise in our design which was associated with spatial navigation, could continuously activate the regions of posterior hippocampal covariance network. As a result, the distinct patterns of anterior and posterior hippocampal structural covariance in LV3 were both strengthened. These results also suggest that musical theory learning and finger exercise activities involved in musical training could promote the differentiation of the structural covariances of the aHC and pHC. On the other hand, research has confirmed that during memory formation, the hippocampus could play a role according to the memory content, or as a whole, or be divided into different parts responsible for different functions (Moser and Moser, 1998; Nadel et al., 2013). These conclusions indicate that the differentiation of the hippocampal structural covariance along the longitudinal axis is to meet the needs of the brain to achieve different cognitive functions, and an enhanced degree of this differentiation may have a positive effect on improving the cognitive ability of the brain. Our results provide new ideas for understanding the role of musical training in improving brain cognitive ability.

Our work still has some limitations. First, it is worthwhile evaluating functional network changes to further explore musical training effects. Nevertheless, the structural covariance patterns observed here achieve high similarity with previous findings (Persson et al., 2014; Stening et al., 2017; Nordin et al., 2018), together with the fact that the coordinate-based method used here for anterior and posterior segmentation of the hippocampus has proven to be effective in other studies, speaking to the validity of our results. Second, there were no significant cognitive differences between these two groups before and after musical training. This might be due to the fact that changes of cognition were relatively subtle and difficult to detect using conventional methods for young adults, who had relatively strong cognitive ability, visual processing, and motor abilities. More sensitive behavior measurement would be meaningful to observe the subtle change of behavior induced by musical training in young adults for further analysis. Third, the evaluation of musical performance at the end of training is qualitative. In future, the measures of musical performance deserves to be recorded quantitatively to investigate the relationship between our experimental variables and the musical performance. Fourth, aside from studying the difference in structural covariances induced by musical training along the hippocampal anterior-posterior, high resolution MRI encouraged dividing the hippocampus into cross-sectional subfields to explore their whole-brain structural covariances (Yushkevich et al., 2015; Wang et al., 2018). Previous studies suggested that high accuracy for most subfields can be obtained by using high resolution (e.g. 7T) MRI (Wisse et al., 2016), and a slice thickness of 1 mm or less is needed for accurate segmentation of hippocampal subfields (Malykhin et al., 2010; Bonnici et al., 2012; Malykhin et al., 2017). Therefore, the segmentation of hippocampal subfields using standard resolution T1 images must be cautiously used in this study. In the future, high resolution images should be obtained to explore the impact of musical training on the hippocampal subfields.

In summary, we found that musical training could induce changes in structural covariance of the left aHC and lead the expressed patterns of both anterior and posterior hippocampal whole-brain structural covariance to a greater extent. Our results also supported the functional lateralization of the hippocampus and suggested that the beneficial effects induced by musical training on cognitive ability, might partly be attributed to the greater differentiation of the hippocampal structural covariance network along the longitudinal axis.

## Data Availability Statement

The datasets generated for this study are available on request to the corresponding author.

## Ethics Statement

The studies involving human participants were reviewed and approved by the Biological and Medical Ethics Committee of Beihang University. The patients/participants provided their written informed consent to participate in this study.

## Author Contributions

PG analyzed the data, prepared the figures and drafted the manuscript. PG, QL, and SL interpreted the results of experiments, edited, and revised the manuscript. All authors contributed to writing the manuscript or performing the experiments.

## Conflict of Interest

The authors declare that the research was conducted in the absence of any commercial or financial relationships that could be construed as a potential conflict of interest.

## Footnotes

1 http://www.neuro.uni-jena.de/cat/

2 http://www.fil.ion.ucl.ac.uk/spm/

3 http://fmri.wfubmc.edu/software/PickAtlas

4 http://www.rotman-baycrest.on.ca/pls

## Abbreviations

aHC: anterior hippocampus
AMMA: advanced measures of music audiation
BDI: beck depression inventory
BSR: bootstrap ratio
FDR: false discovery rate
GMV: gray matter volume
IQ: intelligence quotient
LVs: latent variables
pHC: posterior hippocampus
PLS: partial least squares
SDs: standard deviations
TIV: total intracranial volume
TP1: time point 1
TP2: time point 2
TP3: time point 3.
